# Inhibition of PI3K Signalling Selectively Affects Medulloblastoma Cancer Stem Cells

**DOI:** 10.1155/2015/973912

**Published:** 2015-10-18

**Authors:** Chiara Frasson, Elena Rampazzo, Benedetta Accordi, Giacomo Beggio, Francesca Pistollato, Giuseppe Basso, Luca Persano

**Affiliations:** ^1^Istituto di Ricerca Pediatrica Città della Speranza (IRP), Corso Stati Uniti 4, 35127 Padova, Italy; ^2^Department of Woman and Child Health, University of Padova, Via Giustiniani 3, 35128 Padova, Italy; ^3^Department of Neuroscience, University of Padova, Via Giustiniani 2, 35128 Padova, Italy

## Abstract

Medulloblastoma is the most common malignant brain tumor of childhood. Although survival has slowly increased in the past years, the prognosis of these patients remains unfavourable. In this context, it has been recently shown that the intracellular signaling pathways activated during embryonic cerebellar development are deregulated in MDB. One of the most important is PI3K/AKT/mTOR, implicated in cell proliferation, survival, growth, and protein synthesis. Moreover, a fraction of MDB cells has been shown to posses stemlike features, to express typical neuronal precursor markers (Nestin and CD133), and to be maintained by the hypoxic cerebellar microenvironment. This subpopulation of MDB cells is considered to be responsible for treatment resistance and recurrence. In this study, we evaluated the effects of PI3K/AKT pathway inhibition on primary cultures of MDB and particularly on the cancer stem cell (CSC) population (CD133^+^). PI3K inhibition was able to counteract MDB cell growth and to promote differentiation of stemlike MDB cells. Moreover, PI3K/AKT pathway suppression induced dramatic cell death through activation of the mitochondrial proapoptotic cascade. Finally, analysis on the stem cells fraction revealed that the MDB CSC population is more sensitive to PI3K targeting compared to the whole cancerous population and its nonstem cell counterpart.

## 1. Introduction

Medulloblastoma (MDB) is the most frequent primitive neuroectodermal tumor in children. WHO classification of central nervous system tumours subdivides MDB into five histological groups: classic, desmoplastic, MDB with extensive nodularity, anaplastic, and large cell MDB [1]. It has been suggested that these tumours arise from foetal/embryonic tissues as a consequence of deregulated developmental processes [2, 3]. In this context, recent studies have identified four molecular subtypes of MDB tumours depending on the activation of specific embryonic developmental pathways which are, in particular, Wnt subgroup, characterized by somatic mutations occurring in the CTNNB1 gene [4–6], Sonic hedgehog (SHH) subgroup, mainly characterized by the loss of the SHH receptor Patched 1 (PTCH1) [4], a third subgroup (named Group 3) particularly enriched for MYC (c-Myc) gene amplifications, and subgroup four (named Group 4), in which tumours often possess amplification at the level of MYCN and CDK6 genes [7, 8]. The Phosphoinositide-3-Kinase (PI3K)/AKT signalling pathway has often been reported to be deregulated in MDB, with numerous genetic alterations involving this network occurring independently of the particular subtype [8]. Indeed, it has been suggested that many components of this pathway are involved in MDB proliferation, chemoresistance, and metastasis [9–11].

We and others previously supported the existence of a “cancer stem cell” subpopulation (CSC) in brain tumours, including MDB, expressing phenotypic markers generally associated to neural stem cells in the developing brain, such as CD133 and Nestin. These CSCs possess the ability to form neurosphere* in vitro* and to be tumorigenic when xenotransplanted in recipient mice [12–15]. Moreover, recent studies highlighted the role of PI3K/AKT/mTOR pathway in the maintenance and survival of CSCs in solid tumours such as prostate and breast cancers [16, 17]. A potent and selective dual inhibitor of mTORC1/2 and class I PI3-kinases are able to inhibit proliferation and survival of breast CSCs* in vivo* and to markedly reduce their tumor-initiating ability in limiting dilution assays [18]. For all these considerations, we hypothesized that PI3K could be a good target in MDB and particularly MDB CSCs.

In this study, we pharmacologically inhibited PI3K in primary MDB-derived cells showing that the AKT/mTOR network is fundamental for the maintenance of MDB cell proliferation and survival. Moreover, we demonstrated that PI3K inhibition yielded to MDB cell death by specifically affecting the CSC population (CD133^+^), while sparing more differentiated cells, through the activation of the mitochondrial apoptotic cascade.

## 2. Materials and Methods

### 2.1. Isolation and Gas-Controlled Expansion of Cells

Written informed consent for the donation of tumor brain tissues was obtained from parents prior to tissue acquisition, under the auspices of the protocol for the acquisition of human brain tissues obtained from the Ethical Committee board of the University of Padova and Padova Academic Hospital. All tissues were acquired following the tenets of the Declaration of Helsinki. MDB precursors were derived from 3 tumors taken at surgery (see Supplementary Table 1 in Supplementary Material available online at http://dx.doi.org/10.1155/2015/973912); initial pathological review was followed by secondary neuropathological review to reconfirm diagnosis. We dissociated and cultured cells as previously described [19] (in HAM'S-F12/DME, Euroclone) with additional BIT9500 (10%, serum substitute, Stem Cell Technologies) and 20 ng/mL epidermal growth factor (EGF, human from R&D Systems), in an atmosphere of 2% oxygen, 5% carbon dioxide, and balanced nitrogen [14]. For continuous expansion, one-half of the medium was replaced every day and cultures were passaged every 7–10 days using TrypLE (Invitrogen). Cells were not cultured for more than 8 passages* in vitro* in order to avoid long-term culture related effects. DAOY, D341, D348, D425, D458, and D556 MDB cell lines were cultured as described for primary cells in an atmosphere of 2% oxygen or 20% oxygen, 5% carbon dioxide, and balanced nitrogen. PI3K/AKT inhibition was obtained by LY294002 administration to MDB cell media for 24, 48, or 72 hours (h) at 15 *μ*M (Sigma Aldrich). In some experiments, MDB cell lines have been treated for 24 or 48 h with LY294002 and then plated in Methocult semisolid medium (Stem Cell Technologies) at 1000 cells/P12 well.

### 2.2. Flow Cytometry and Cell Sorting

#### 2.2.1. Annexin/PI Staining

Cells were incubated for 15 minutes with Hepes buffer containing anti-Annexin-V-FITC antibody and Propidium Iodide (PI), following manufacturer's instructions (Human Annexin-V-FLUOS Staining Kit, Roche). Relative percentages of different subpopulations were calculated by considering the entire cell population.

#### 2.2.2. CD133 and CD15 Staining

Cells (2 × 10^5^ cells/mL) were incubated with antihuman CD133 (clone AC133/2-PE, 1 : 20, Miltenyi Biotec) or antihuman CD15 (FITC conjugated, 1 : 20, Immunotech) as previously described [14]. Viability was assessed by adding 7-aminoactinomycin-D (7-AAD, 50 ng/mL, BD Bioscience) prior to analysis. Relative percentages of different subpopulations were calculated based on live gated cells (as indicated by physical parameters, side scatter and forward scatter, and negativity for 7-AAD). Unlabeled cells and cells incubated with appropriate isotype control antibodies were first acquired to ensure labelling specificity. For both stainings, cells were analyzed on a Cytomics FC500 flow cytometer (Beckman Coulter).

#### 2.2.3. CD133 Cell Sorting

In cell sorting experiments, MDB cells were analyzed and then sorted on the basis of CD133 expression. A CD133 versus side scatter dot plot revealed the populations of interest that were sorted: CD133^+^ and CD133^−^ cell fractions were selected by setting appropriate sorting gates as previously described [20].

### 2.3. Reverse-Phase Protein Arrays (RPPA)

Cells were washed with ice-cold PBS 1X and lysed on ice for 20 min in an appropriate lysis buffer: TPER Reagent (Pierce), 300 mM NaCl, 1 mM Na-orthovanadate, 200 mM PEFABLOC (AEBSF) (Roche), 1 *μ*g/mL Aprotinin (Sigma Aldrich), 5 mg/mL Pepstatin A (Sigma Aldrich), and 1 mg/mL Leupeptin (Sigma Aldrich). Cell lysates were then cleared by centrifugation, and supernatants were collected and assayed for protein concentration with the BCA Protein Assay Reagent Kit (Pierce). Cell lysates were diluted to 1 mg/mL in a mixture of 2x Tris-Glycine SDS Sample Buffer (Invitrogen) plus 5% of *β*-mercaptoethanol. Lysates were stored at −80°C and boiled for 8 min immediately prior to arraying.

Lysates were loaded into a 384-well plate and serially diluted with lysis buffer into four-point dilution curves. As positive controls for antibody staining, we added also the three commercial cell lysates Hela+Pervanadate, A431+EGF, and Jurkat Apoptotic (BD Biosciences). Samples were printed in duplicate onto nitrocellulose-coated slides (FAST slides, Whatman Schleicher & Schuell) with the 2470 Arrayer (Aushon BioSystems).

One slide was stained with Fast Green FCF dye (Sigma Aldrich) according to the manufacturer's instruction, in order to estimate the total protein amount of each printed sample. Before antibody staining, the arrays were blocked for 3 h at room temperature in blocking solution (2 g I-block and 0.1% Tween-20 in 1 L of PBS 1X). Blocked arrays were stained with the following primary antibodies on an automated slide stainer (Dako Autostainer Plus, Dako Cytomation) using the CSA kit (Dako Cytomation) as described previously [21]: AKT (S473) (1 : 100), AKT (T308) (1 : 100), PDK1 (S241) (1 : 100), IRS-1 (S612) (1 : 50), 4EBP1 (S65) (1 : 250), eIF4G (S1108) (1 : 100), and GSK3*α* (S21) (1 : 50) (all from Cell Signaling Technology). Slides were air-dried and scanned on Epson Perfection V300 Photo at 600 dpi. For an example of antibody-stained slides, please see Supplementary Figure 1.

Each antibody was previously subjected to extensive validation for single-band specificity by Western blot. An antibody has been considered validated for RPPA staining if showing a single band at the right molecular weight in lysates of control cell lines (not overexpressing cell lysates) and of tissue specimens similar to that printed on RPPA slides, extracted with the same lysis buffer.

The TIF images of antibody- and Fast Green FCF-stained slides were analyzed using Microvigene Software (VigeneTech Inc.) to extract numeric intensity values from the array images, as described [21].

### 2.4. Western Blot

Total protein extracts were isolated in lysis buffer as previously described [22]. Equal amounts of proteins (10–20 *μ*g) were resolved using SDS-PAGE gels and transferred to PVDF Hybond-p membrane (GE Healthcare). Membranes were blocked with I-block (Life Technologies) for at least 2 hours, under rotation at RT. Membranes were then incubated overnight at 4°C under constant shaking with the following primary antibodies: HIF-1*α* (mouse, 1 : 250, BD Pharmingen), mTOR total, mTOR (S2448), P70S6K total, P70S6K (T389), AKT total, AKT (T308), AKT (S473), BAX, PARP, (all rabbit, 1 : 1000, Cell Signaling Technologies), and *β*-actin (mouse, 1 : 10000, Sigma Aldrich) as loading control. Membranes were next incubated with peroxidase-labeled goat anti-rabbit IgG or goat anti-murine IgG (both 1 : 50.000 in I-block from Sigma Aldrich) for 60 min. All membranes were visualized using ECL Select (GE Healthcare) and exposed to Hyperfilm MP (GE Healthcare).

### 2.5. Immunofluorescence and TUNEL Assay

Cells were fixed in cold 4% formaldehyde for 15 min, rinsed, and stored at 4°C prior to analysis. Primary antibody staining was performed with Ki67 (mouse, 1 : 100, Dako), Nestin (mouse, 1 : 200, Millipore), and *β*-III-tubulin (Tuj-1, mouse, 1 : 1000, Covance). After incubation, cells were washed and incubated with specie-specific secondary antibodies conjugated to Alexa dyes (Invitrogen).

Apoptotic DNA fragmentation was analyzed by TUNEL assay (terminal deoxynucleotidyl transferase-mediated dUTP nick end labeling) by using the* In Situ* Cell Death Detection Kit (Roche Diagnostics) according to manufacturer's instructions.

In all experiments, cell nuclei were counterstained with DAPI (1 : 10000, Sigma Aldrich), with staining being visualized by epifluorescence (Vico, Nikon) and images compiled for figures using Adobe Illustrator (Adobe).

### 2.6. Statistical Analysis

Graphs and statistical analyses were prepared using Prism 4.00 (Graph Pad). All values are presented as mean ± standard error of the mean (S.E.M.). Statistical significance was measured by one-way ANOVA with Newman-Keuls multiple comparison post Test or *t*-test depending on the comparison: ^*∗*^ *P* < 0.05, ^*∗∗*^ *P* < 0.01, and ^*∗∗∗*^ *P* < 0.001. For all graphs, an asterisk above a bar indicates a significant difference with control untreated cells or with another variable as indicated in the corresponding figure legend.

## 3. Results

### 3.1. PI3K Inhibition Counteracts Proliferation and Induces Apoptosis in MDB Cell Lines

**Figure 1:**
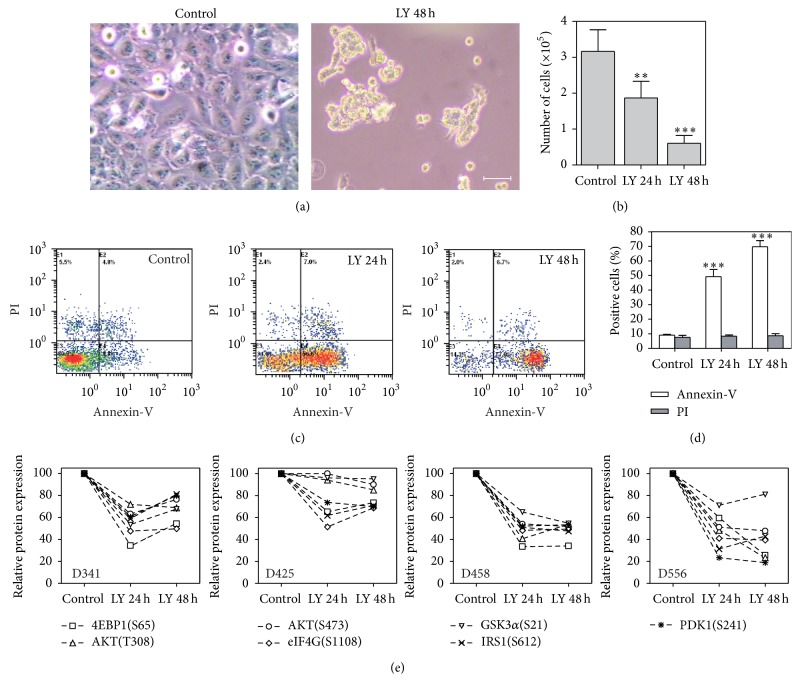
LY294002 reduces cell proliferation and vitality of MDB cell lines by efficiently inhibiting PI3K/AKT signalling. (a) Representative images of DAOY MDB cells after 48 h of treatment with 15 *μ*M LY294002 (LY) compared to control (DMSO). Original magnification 20x, white bar: 50 *μ*m. (b) Bar graph reporting Trypan Blue cell counts of DAOY cells after 48 h of 15 *μ*M LY294002 treatment. Mean of 3 experiments ± S.E.M. (c, d) Representative dot plot showing Annexin-V/PI staining of DAOY cells after 15 *μ*M LY294002 treatment (c) and bar graph summarizing Annexin-V/PI analysis derived from 3 independent experiments ± S.E.M (d). (e) Graphs showing relative expression of the PI3K/AKT signalling components (phosphorylated at specific residues) 4EBP1, AKT, eIF4G, GSK3*α*, IRS1, and PDK1 as measured by RPPA. For all graphs, ^*∗∗*^ *P* < 0.01 and ^*∗∗∗*^ *P* < 0.001.

The PI3K/AKT axis has been recently reported as a critical signalling pathway in the maintenance of human MDB tumour growth, metastasis, and chemoresistance [9–11]. For this reason, we first treated MDB cell lines with the PI3K/AKT inhibitor LY294002 for 24 or 48 hours (h) and evaluated the effects on cell proliferation and apoptosis. PI3K inhibition induced dramatic morphological changes in DAOY treated MDB cells, promoting cell detachment from the plate and clumps formation (Figure 1(a)). In parallel with these morphological changes, cell count significantly decreased starting from 24 h from LY294002 treatment and further diminished after 48 h (Figure 1(b)). To further confirm these data, we treated with LY294002 also D341, D348, D425, D458, and D556 MDB cell lines which all underwent a dramatic reduction of cell number after treatment at the same time points (Supplementary Figure 2(a)). To give reason of the observed decrease in cell number, we hypothesized that PI3K/AKT inhibition could promote apoptosis in MDB cell lines. To test this hypothesis, we performed Annexin-V/PI staining on DAOY cells treated for 24 and 48 h with LY294002 and analyzed them by flow cytometry. PI3K inhibition mediated by LY294002 was able to dramatically induce cell death in DAOY MDB cells with almost 80% of cells positive for Annexin-V after 48 h from treatment (Figures 1(c) and 1(d)). Accordingly, also D341, D425, D458, and D556 MDB cell lines all showed a significant increase of Annexin-V staining after LY294002 treatment at the same time points (Supplementary Figure 2(b)). In addition, PI3K inhibition mediated by LY294002 treatment was sufficient to counteract the clonogenic ability of DAOY, D341, and D425 MDB cell lines as shown in Supplementary Figure 2(c), confirming the importance of PI3K/AKT in supporting MDB cell growth. It is of note that D458 and D556 cell lines were not able to grow as clones; thus they have not been included in this analysis.

To confirm LY294002 effectiveness in abolishing PI3K/AKT signalling activation, we treated four MDB cell lines (D341, D425, D458, and D556) with LY294002 for 24 and 48 h, collected cell lysates, and then performed Reverse-Phase Protein Array (RPPA) analysis for the expression of a series of PI3K/AKT pathway components. In particular, we evaluated the phosphorylation status of 4EBP1 (S65), AKT (T308), AKT (S473), EIF4G (S1108), GSK3*α* (S21), IRS1 (S612), and PDK1 (S241), demonstrating a general downmodulation of PI3K signalling activation in treated MDB cell lines (Figure 1(e)).

### 3.2. PI3K Inhibition Affects Proliferation of Primary MDB-Derived Cells

**Figure 2:**
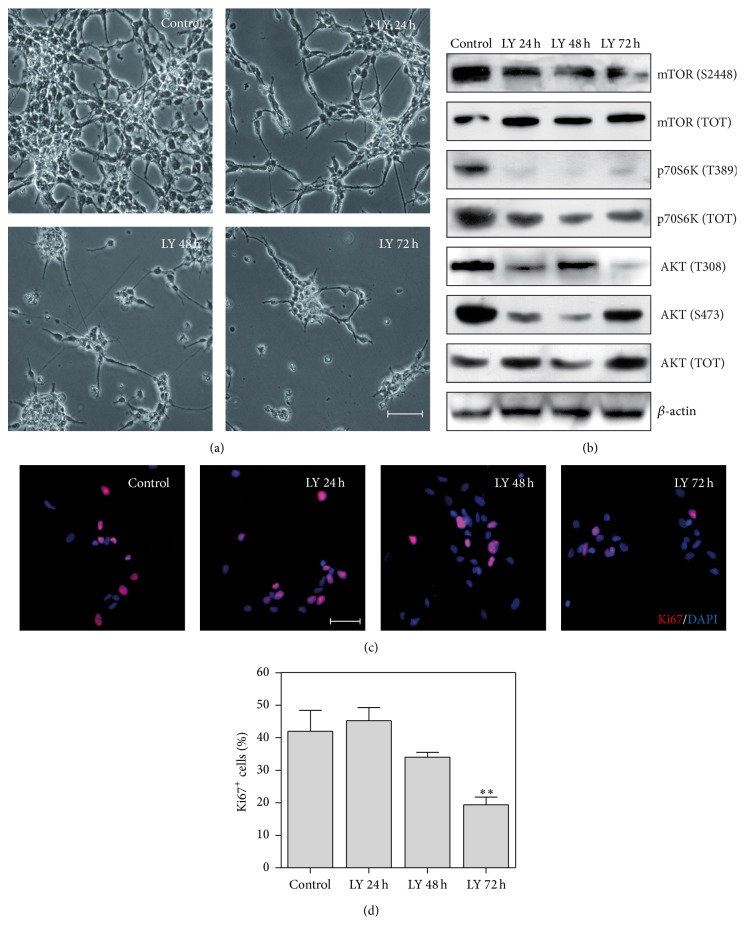
LY294002 inhibits PI3K/AKT signalling and proliferation of MDB patients-derived primary cells. (a) Representative images of HuTuP33 primary MDB cells at different time points from LY294002 (LY) treatment (15 *μ*M). Original magnification 20x, bar: 50 *μ*m. (b) Western blot analysis performed on FI25 primary MDB cells at different time points from LY294002 treatment (15 *μ*M). *β*-actin levels have been used as loading control and protein phosphorylation levels compared to the corresponding total proteins. Similar results have been obtained also for HuTuP33 and HuTuP49 MDB primary cells. (c, d) Representative immunofluorescence images of FI25 cells after LY294002 treatment (15 *μ*M) showing a significant decrease of Ki67 (red) expression (c) and relative quantification (d). Original magnification 10x, bar: 50 *μ*m. Similar images were obtained also for HuTuP33 primary MDB cells. Mean of 3 independent experiments ± S.E.M, ^*∗∗*^ *P* < 0.01.

Starting from these preliminary observations obtained in MDB cell lines, we sought to test the effects of PI3K/AKT inhibition also in MDB patient-derived primary cell cultures. As shown in Figure 2(a), PI3K inhibition was able to strongly impact the cell number of primary MDB cells at the same concentration used for treatment of MDB cell lines. Since primary MDB cells possess an intrinsic lower proliferation rate than MDB cell lines, we extended the analysis of the effects exerted by LY294002 treatment until 72 h after drug exposure. Given the strong effect of LY294002 on cell count, we sought to confirm the efficacy of LY294002 in inhibiting PI3K activation by analyzing the phosphorylation status of its downstream effectors after 24, 48, and 72 h from treatment. Western blot analysis demonstrated a potent downmodulation of the PI3K/AKT signalling pathway starting from 24 h from treatment and showed that LY294002 was able to maintain a general signalling inhibition until 72 h. In particular, a dramatic inhibition of the phosphorylation status of the PI3K direct target AKT occurred at both serine 473, particularly relevant for the regulation of cellular stress response in tumour cells [23], and threonine 308, reported to be involved in the process of glial differentiation of neural stem cells [24]. The AKT target mTOR was also inhibited as shown by a decrease in mTOR phosphorylation at serine 2448 followed by P70S6K downregulation. Indeed, P70S6K is a well described mTOR target protein involved in protein synthesis processes [25]. Surprisingly, we observed a recovery of AKT phosphorylation at serine 473 after 72 h of treatment. Starting from this evidence, we compared this result with RPPA data obtained from MDB cell lines after 72 h of LY294002 treatment and found a general recovery of PI3K/AKT signalling activation in all cell lines tested at this prolonged time point (Supplementary Figure 3). However, this apparent paradox has already been reported in LY294002 treated acute myeloid leukemia (AML) cells. Indeed, despite being effective in lowering PI3K signalling activation in these cells, after prolonged treatment, LY294002 promoted a protein network-based feedback mechanism able to reinduce AKT phosphorylation through the overexpression of RTKs or IRS-1 stabilization [26].

To further confirm the inhibition of cell growth mediated by PI3K/AKT pathway downmodulation, we analyzed the expression of the cell cycle marker Ki67 on LY294002 treated cells. By immunofluorescence analysis, we found a decrease in the number of actively cycling cells (Ki67^+^). Indeed, MDB treated cells decreased Ki67 marker expression, suggesting an exit from the cell cycle to enter in G_0_ phase starting from 48 h after PI3K inhibition, to be significantly decreased at 72 h after LY294002 treatment (Figures 2(c) and 2(d)).

### 3.3. PI3K/AKT Inhibition Induces Apoptosis in Primary MDB-Derived Cells

To better explore the mechanisms underlying the strong decrease of MDB cell number, we investigated if PI3K inhibition could bring primary MDB cells to apoptosis, as previously shown for MDB cells (Figures 1(c) and 1(d) and Supplementary Figure 2(b)).

**Figure 3:**
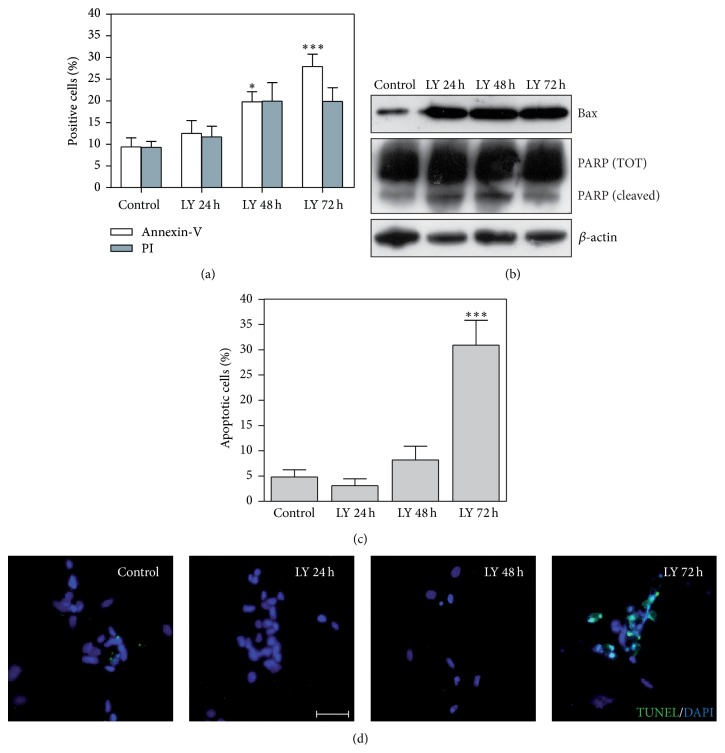
PI3K/AKT inhibition induces strong apoptosis in MDB primary cells. (a) Bar graph reporting data from Annexin-V/PI analyses performed on primary MDB cells (HuTuP33, HuTuP49, and FI25) after LY294002 (LY) treatment (15 *μ*M). (b) Western blot analysis performed on FI25 primary MDB cells showing a dramatic induction of the apoptotic process after LY294002 treatment. *β*-actin levels have been used as loading control. (c, d): TUNEL (green) cell death analysis performed on MDB primary cells (FI25 and HuTuP33), showing a significant increase of cell death after 72 h of LY294002 treatment (c) and relative quantification (d). Original magnification 20x, bar: 50 *μ*m. For all graphs, values are represented as mean of at least 3 independent experiments ± S.E.M. ^*∗*^ *P* < 0.05 and ^*∗∗∗*^ *P* < 0.001.

Firstly, we measured a progressive, strong, and significant increase of the Annexin-V^+^/PI^+^ positive cells during time, in particular, after 72 h from PI3K inhibition with LY294002, compared to control cells (Figure 3(a)).

Cell death induction was accompanied by a dramatic overexpression of BAX (Figure 3(b) upper line). BAX is a proapoptotic protein regulated by PI3K/AKT signalling activation whose translocation to mitochondria activates the mitochondrial-mediated apoptotic pathway. Indeed, it has been recently reported that BAX is phosphorylated at serine 184 by AKT and this phosphorylation contributed to the suppression of Bax-mediated death of neutrophils [27]. Moreover, PARP cleavage strongly increased starting from 24 h from treatment (Figure 3(b) intermediate line), indicative of the activation of the mitochondrial apoptotic pathway with release of cytochrome C, caspases activation, and its proteolytic cleavage. The role of PARP in the apoptotic cascade is to promote apoptosis by preventing DNA repair-induced survival [28]. Thus, to finally confirm the “mitochondrial apoptosis hypothesis,” we performed TUNEL analysis on LY294002 treated cells. In line with previous results, as a consequence of PI3K inhibition, we measured a significant number of TUNEL^+^ nuclei at 72 h from LY294002 treatment (Figures 3(c) and 3(d)). All these data support that PI3K inhibition strongly induces the activation of the apoptotic cascade and that this cell death program is mediated by mitochondrial mechanisms.

### 3.4. LY294002 Treatment Promotes Neuronal Differentiation of Primary MDB Cells

Starting from the evidence of reduction of proliferation and induction of cell death, we then wonder about the phenotypic identity of the remaining living cells.

**Figure 4:**
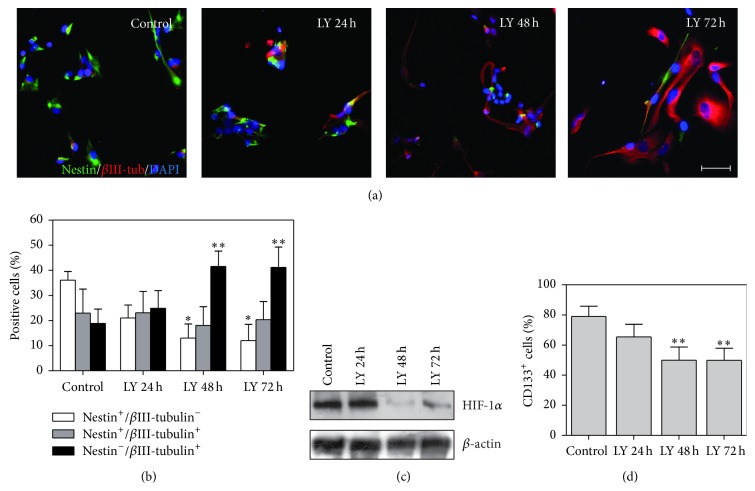
PI3K/AKT inhibition promotes neuronal differentiation of MDB primary cells. (a, b): representative images (FI25) (a) and relative quantification (FI25 and HuTuP33) (b) of percentages of cells positive for Nestin (green) and/or *β*III-tubulin (red) at different time points from LY294002 (LY) treatment. Original magnification 20x, bar: 20 *μ*m. (c) Western blot analysis reporting HIF-1*α* levels after PI3K/AKT inhibition mediated by LY294002. (d) Bar graph showing CD133^+^ cells decrease after LY294002 treatment (HuTuP33, HuTuP49, and FI25). For all graphs, mean of 3 independent experiments ± S.E.M. ^*∗∗*^ *P* < 0.01.

We analyzed specific neural markers expression to unveil the differentiation status of LY294002 treated cells. Immunofluorescence analysis revealed a progressive phenotypical change in MDB tumour cells after 24, 48, and 72 h treatment. In particular, after 72 h of LY294002 exposure, we measured an almost complete loss of the neural stem cell marker, Nestin, with cells acquiring a strong expression of the neuronal marker, *β*-III-tubulin, compared to control cells (Figures 4(a) and 4(b)). Moreover, an evident reduction of HIF-1*α* expression that we previously described to be a hypoxia and stem cell marker in MDB [14] occurred as a consequence of PI3K pathway inhibition, as shown by Western blot analysis in Figure 4(c).

To further decipher the relationship between MDB cell phenotype and PI3K inhibition, we then analyzed the expression of the neural stem cell marker CD133, previously shown to be a suitable marker for brain tumour stem cells, including MDB [14, 29]. Cytofluorimetric analysis of CD133 expression evidenced a progressive and significant decrease of the CD133^+^ cancer stem cell subpopulation particularly at 48 and 72 h after treatment, compared to control cells (Figure 4(d)). Another marker, described to be indicative of stemness in MDB murine models, is CD15 [30]. We found only a poor expression of CD15 in our human model of MDB-derived cells (data not shown), thus excluding this marker from further analyses.

### 3.5. MDB CSCs Are More Sensitive to PI3K Inhibition-Induced Cell Death

Since recent studies pointed out the CSC subpopulation in tumours (including brain tumours) as the leading driver of malignant progression and relapse [31] and previous results reported in this study clearly showed that PI3K/AKT downmodulation led to MDB cell growth inhibition, induction of cell death, and cellular differentiation, we examined the possible relationship between differentiation and cell death in MDB primary cells. In this context, we hypothesized that PI3K/AKT inhibition could preferentially target the stem cell fraction (CD133^+^ and/or Nestin^+^) of MDB cells, thus provoking their cell death while sparing the more differentiated fraction.

**Figure 5:**
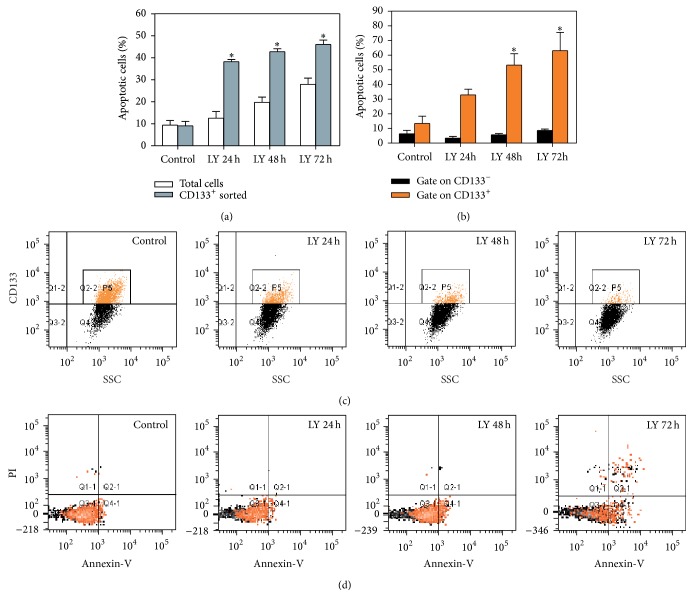
PI3K/AKT inhibition induces significant cell death selectively in CD133^+^ MDB primary cells. (a) Bar graph comparing LY294002- (LY-) induced cell death between CD133^+^ MDB primary cells (sorted) and the total cell population by Annexin-V staining. CD133^+^ cells demonstrate a significant (^*∗*^ *P* < 0.05) increase of apoptosis levels. (b–d) Cytofluorimetric analysis of MDB primary cells showing CD133^+^ cell subpopulation (orange label) is more sensitive to LY294002 treatment than CD133^−^ cells (black label) as shown by Annexin-V staining in (d). Representative dot plots are reported for cytofluorimetric analysis of HuTuP33 MDB cells. For all graphs, mean of 3 independent experiments ± S.E.M. ^*∗*^ *P* < 0.05 in the comparison with control cells.

In order to investigate the effects of PI3K inhibition mediated by LY294002 specifically in the stem cells compartment, we sorted primary MDB cells for their expression of CD133 by FACS and then treated them with LY294002 for 24, 48, and 72 h. As reported in Figure 5(a), CD133^+^ cells resulted to be more sensitive to PI3K inhibition compared to the total MDB cell population, showing a progressive and significant increase in the Annexin-V^+^ cell fraction during time. Cell sorting is a highly stressful procedure which can eventually interfere with cell survival particularly when analyzing stress sensitive cells as primary MDB-derived cells [14]. For this reason, to more accurately measure induction of cell death in specific cellular populations, we stained MDB primary cells with both Annexin-V and CD133 antibody, to directly evaluate the coexpression of these two markers. This analysis confirmed that CD133^+^ MDB cells progressively diminished during time (Figures 4(d) and 5(c)), being more sensitive to PI3K/AKT inhibition, as shown by a dramatic increase in the number of CD133^+^/Annexin-V^+^ cells (up to 82% after 72 h) (Figures 5(b)–5(d), orange label). Conversely, the nonstem CD133^−^ cell fraction did not show any induction of cell death when compared to control untreated cells at the same time points of treatment (Figures 5(b)–5(d), black label). In summary, this analysis demonstrates that the most aggressive MDB subpopulation (stem cells) is the preferential target of PI3K inhibition mediated by LY294002, highlighting the relevance of PI3K/AKT signalling as therapeutic target for MDB.

## 4. Discussion

MDB represents the 20% of pediatric brain tumours, peaking in incidence between 4 and 7 years [32]. Although prognosis has significantly improved in the last decades with multimodal therapy which includes surgery, radiotherapy, and chemotherapy, one-third of patients still succumb to their disease [33, 34]. Moreover, the outlook for patients with metastatic or recurrent disease remains poor, with posttreatment sequelae often resulting in significant long-term intellectual and/or developmental impairments [35]. For these reasons, further research is needed to find more efficient treatment strategies for prognostically unfavourable patient groups.

Since alterations in the activation status of PI3K/AKT pathway have been described to dramatically impact cancer cell proliferation, migration, and, in general, its aggressiveness [32], in this study, we evaluated the effects mediated by the specific inhibition of this signalling in both MDB cell lines and patient-derived primary cultures. Hartmann et al. demonstrated that cell proliferation was clearly correlated to AKT phosphorylation (S473) in a series of MDB tumours while adjacent normal cerebellar tissues were characterized by lower levels of AKT activation [11]. Moreover, PI3K/AKT axis is able to enhance the intracellular signalling of SHH in neural precursors [36] and MDB-derived cells, in which it has often been found to be deregulated [4, 37, 38]. Here, we demonstrated that PI3K inhibitor LY294002 is able to suppress PI3K/AKT signalling, thus counteracting MDB cell proliferation and inducing mitochondrial cell death in primary MDB-derived cells.

As described also for other brain tumours, the “stem cells origin” of cancer cells has been proposed also for MDB [15, 39]. In particular, a great similarity between stem cells residing in the ventricular zone of the fourth ventricle, external granule layers progenitor cells, and MDB-derived cells has recently been reported [39, 40]. In addition, other cerebellar precursors have been described to potentially give rise to MDB tumours in particular conditions. Lee et al. characterized a population of CD133^+^ stem cells concentrated in the white matter of the normal postnatal cerebellum [41]. Indeed, other authors identified novel precursors in the upper rhombic lip capable of generating Pax6/Tbr2/Tbr1 and Math1 expressing cells in the deep cerebellar white matter [42, 43]. It is of note that these markers have often been found to be expressed in different MDB subtypes [44, 45].

In the recent years, the so-called CSCs are becoming the most attractive target for experimental therapies. Indeed, CSCs have been described not only to be responsible for tumors progression but also (in particular for brain tumour CSCs) to be able to circumvent standard chemoradiotherapy, causing relapse. In line with these considerations, it has been previously reported that one of the leading pathways involved in the regulation of embryonic stem cell differentiation and resistance of MDB CSCs to therapy is PI3K/AKT signalling [46, 47]. Accordingly, Ehrhardt et al. recently demonstrated that PI3K inhibition had antiproliferative and proapoptotic effects in MDB cell lines. Moreover, they showed that PI3K inhibition is able to attenuate the migratory capacity of MDB cells lines and to target the stemlike subpopulations expressing the stem cell marker CD133 [48].

Importantly, here we demonstrated that PI3K inhibition is able to selectively target the CSC subpopulation (CD133^+^) also in MDB primary cells, sparing its most differentiated cellular counterpart. Moreover, since we found strong neuronal differentiation associated with PI3K inhibition (Figure 4), we hypothesized that these differentiated cells could be nonstem cells (CD133^−^) which, being insensitive to PI3K inhibition (Figure 5), had escaped treatment.

Since only CD133^+^ cells were capable of generating MDB xenografts phenotypically identical to the original human tumors [15], these cells should be the primary target of therapy, in particular, by combination strategies. In addition, Ong et al. demonstrated that high expression of CD133 is associated with resistance of CSCs to 5-FU-based chemotherapy as well as with a significant worse survival in a colon cancer model [49]. As a result, we recently demonstrated that a specific targeting of the stem cell compartment in brain tumours can boost standard chemotherapy efficacy [50]. These considerations underline the importance of designing new combinatorial therapeutic strategies aimed to impair both the survival machinery of CSCs and tumour bulk cells.

Our results are particularly relevant for their possible impact on the future development of CSC-targeted therapies, with the final aim of halting the relevant pathways involved in their survival.

## 5. Conclusions

In conclusion, in this study, we demonstrate that PI3K signalling is one of the most critical pathways sustaining MDB cell growth and survival. Indeed, suppression of PI3K activation mediated by the inhibitor LY294002 is able to dramatically impact MDB cell proliferation and to induce the activation of the mitochondrial cell death program. CSCs have been reported to be one of the driving forces of tumour progression and resistance to therapy, also in MDB. For this reason, we evaluated the effects mediated by PI3K inhibition also in MDB CSCs identified by CD133 expression. Surprisingly, we found CD133^+^ MDB cells to be more sensitive to PI3K signalling suppression rather than the entire MDB cell population or their CD133^−^ counterpart. These results support once again the hypothesis that a CSC-targeted treatment should strongly counteract the aggressive features of cancerous cells. Indeed, if CSCs are the key feeders of the bulk cancerous population in a tumour, their sparing during treatment would inevitably bring to recurrence. Therefore, the future development of more effective treatments for MDB would necessarily take into account the effects exerted to these stemlike populations and the possible design of CSC-tailored therapeutic strategies.

## Supplementary Material

Supplementary Material contains a Table containing information about the primary cell lines used in this study and figures with data supporting manuscript claims.
