# Supplementary material for: Inhibition of PI3K Signalling Selectively Affects Medulloblastoma Cancer Stem Cells
Source: Biomed Res Int. 2015 Oct 18;2015:973912. doi: 10.1155/2015/973912 (PMC4628705; doi:10.1155/2015/973912)

Submitted to the Special Issue: "Phosphorylation, Signaling, and Cancer: Targets and Targeting"

SUPPLEMENTARY INFORMATION

Supplementary Table 1. MDB-derived primary cells used in this study.

**Code**	**Tumour Type**	**Age (years)**	**Gender**
HuTuP33	Medulloblastoma	1	Female
HuTuP49	Medulloblastoma	4	Female
F125	Medulloblastoma	1	Male

SUPPLEMENTARY FIGURE LEGENDS

Supplementary Figure 1. RPPA stained slide. Representative RPPA slide stained for eIF4G(S1108) antibody (Cell Signaling Technologies) at 1:100 dilution.

Supplementary Figure 2. PI3K inhibition reduces cell number and clonogenic ability of MDB cell lines. (**A**) Bar graph summarizing cell number reduction of LY294002 (LY)-treated MDB cell lines including D341, D384, D425, D458 and D556. Mean of at least 4 independent experiments ± S.E.M. (**B**) Bar graph summarizing Annexin-V/PI analysis derived from 3 independent experiments ± S.E.M. performed on D341, D425, D458 and D556 MDB cell lines. *$p<0.05$, **$p<0.01$, ***$p<0.001$. (C) Representative images showing the impairment of the clonogenic ability mediated by LY294002 treatment for 24 or 48h in DOAY (upper panels), D341 (middle panels) and D425 (lower panels) MDB cell lines.

Supplementary Figure 3. LY294002 treatment inhibits the activation of PI3K/AKT signalling components. Graphs showing relative expression of the PI3K/AKT signalling components (phosphorylated at specific residues) 4EBP1, AKT, eIF4G, GSK3α, IRS1 and PDK1 as measured by RPPA until 72 hours of treatment.

Supplementary Figure 1

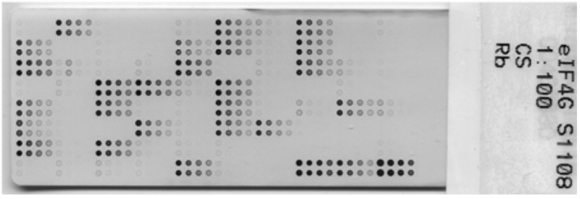

Supplementary Figure 2

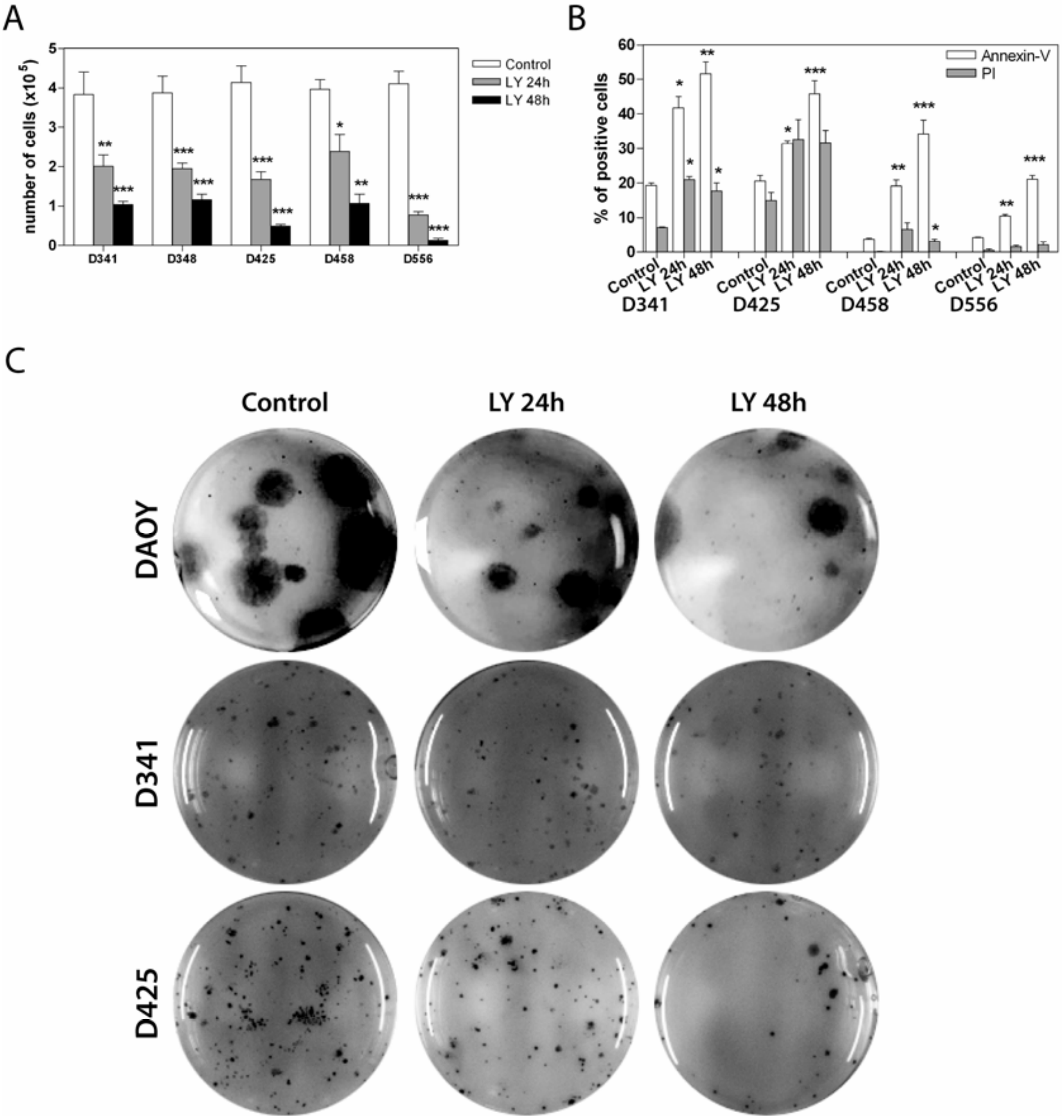

Supplementary Figure 3

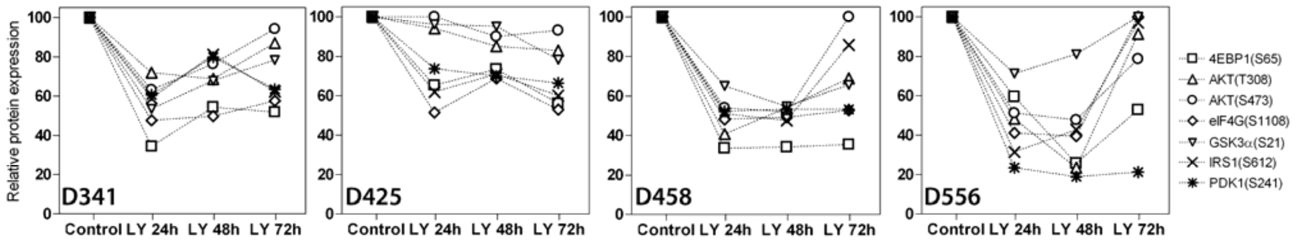

Supplement: Supplementary file 1 — Supplementary Material contains a Table containing information about the primary cell lines used in this study and figures with data supporting manuscript claims. [file 973912.f1.pdf]
